# THE ROLE OF WISDOM IN ADAPTING TO LATE-LIFE SPOUSAL LOSS

**DOI:** 10.1093/geroni/igad104.0396

**Published:** 2023-12-21

**Authors:** Kiana Cogdill-Richardson, Susan Bluck

**Affiliations:** University of Florida, Gainesville, Florida, United States; University of Florida, Gainesville, Florida, United States

## Abstract

Death of one’s spouse is normative but one of life’s most challenging events (Carr et al., 2001). Wisdom involves compassion, tolerance for uncertainty and reflection, so may be particularly useful in dealing with spousal loss. Past research has related personal wisdom to well-being and life satisfaction in general (Ardelt, 2019). Almost no research, however, has examined wisdom as a resource in the context of bereavement. We (1) expected that higher wisdom would predict better grief adaptation in widowed older adults and, (2) explored whether wisdom effects might be moderated by older adults’ physical status. Participants (N = 54, Mage = 81; SD = 7.57, 62.3% female) completed the Brief Wisdom Screening Scale (Glück et al., 2013) and two assessments of grief adaptation: Inventory of Complicated Grief (Prigerson et al., 1995) and Integration of Stressful Life Experiences (Holland et al., 2011). Hierarchical regression, F(2, 51) = 4.25, p <.05, showed greater wisdom, β = -.27, t(52) = -2.061, p < .05 and higher physical functioning (β = -.27) both predict lower symptoms of complicated grief, with no moderation. A second regression, F(2,51) = 4.44, p < .055, showed a similar trend with greater wisdom associated with better integration of loss, β = .46, t(52) = 1.96, p = .055. Though preliminary, these findings suggest wisdom may serve as a resource for individuals facing loss in later life. Results are discussed in terms of theoretical models (Glück & Bluck, 2013) identifying reciprocal relations between wisdom development and life challenges.

